# Prevalence and associated risk factors of violence against conflict–affected female adolescents: a multi–country, cross–sectional study

**DOI:** 10.7189/jogh.07.010416

**Published:** 2017-06

**Authors:** Lindsay Stark, Khudejha Asghar, Gary Yu, Caroline Bora, Asham Assazenew Baysa, Kathryn L Falb

**Affiliations:** 1 Department of Population and Family Health, Columbia University, New York, NY, USA; 2 New York University College of Nursing, New York, New York, USA; 3 International Rescue Committee, New York, New York, USA

## Abstract

**Background:**

Over half of displaced civilians in humanitarian emergencies are children, and these settings pose unique threats to children’s safety with long–lasting consequences. Our study broadens the limited evidence on violence against adolescent girls in emergencies by estimating prevalence and predictors of violence among adolescent girls aged 13–14 in South Kivu, Democratic Republic of the Congo (DRC), and aged 13–19 in refugee camps in the Benishangul–Gumuz region of Ethiopia.

**Methods:**

Survey data were collected from a sample of 1296 adolescent girls using Computer–Assisted Personal Interview and Audio Computer–Assisted Self–Interview programming. Predictors of violence were modeled using multivariable logistic regression.

**Results:**

The majority of adolescent girls (51.62%) reported experiencing at least one form of violence victimization in the previous 12 months: 31.78% reported being hit or beaten, 36.79% reported being screamed at loudly or aggressively, and 26.67% experienced unwanted sexual touching, forced sex, and/or sexual coercion. Across both countries, ever having a boyfriend and living with an intimate partner were strong predictors of violence. Fewer years of education completed in DRC, and young age in Ethiopia, were also associated with reported victimization.

**Conclusions:**

Prevalence of violence against adolescent girls is high in these two conflict–affected contexts. Findings indicate a need for programs targeting younger populations, broader efforts to address different forms of victimization, and increased recognition of intimate partners and caregivers as perpetrators of violence in conflict–affected settings.

Violence against women and girls is a global epidemic that affects one in three women and one in four girls under the age of 18 [1,2]. Worldwide, females are at highest risk for violence during adolescence, and violence is the second leading cause of death for adolescent girls aged 10–19 [3,4]. Risks of victimization are amplified by both gender and age: societies that support male dominance and restricted roles of females are associated with greater levels of violence against women, and adolescents have less access to services due to their age [5,6]. Physical, emotional, and sexual violence victimization have been associated with negative health consequences that include increased risk of HIV infection, unintended pregnancy, alcohol and substance abuse, depression, post–traumatic stress disorder, and suicide [7–10].

A number of predictors of violence against women and girls have been identified from developed and developing contexts, largely from studies of intimate partner violence [11,12]. These predictors may best be understood within an ecological framework, which posits that there is no single factor that causes such violence, but that violence is a function of many factors that interact at different levels of the “social ecology” [13]. Indeed, studies have found that demographic predictors of physical, emotional, and sexual violence victimization include factors at the societal level, such as unequal access to wage employment; at the communal level, such as norms that support gender inequity; at the interpersonal level, such as living with a single parent and lower socio–economic status; and at the individual level, such as female gender, lower educational attainment, and younger age [14–19]. Previous studies have also found behavioral risks such as engagement in a relationship that includes sexual intercourse and greater number of romantic partners [10,20]. Additionally, female violence victimization in childhood and adolescence consistently predicts later physical and sexual victimization [21,22].

Humanitarian emergencies pose unique threats to safety, as they may alter family structure, reduce access to basic rights such as health care and education, and increase engagement in risky behaviors. Humanitarian emergencies have resulted in an estimated 59.5 million displaced persons, half of whom are children [23]. Previous research suggests that rates of physical and sexual violence may increase during periods of conflict and that such violence is associated with adverse health outcomes in conflict–affected children [24–29]. Adolescent girls have been shown to be particularly vulnerable to victimization, yet, until recently, have been often overlooked in these contexts [25].

There is limited understanding of both prevalence and predictors of violence against conflict–affected adolescent girls. To date, studies that examine violence in conflicts (eg, communities where at least two armed groups have fought) have focused primarily on the health–related *consequences* of violence exposure and on females over the age of 15 [24,30–32]. Large multi–country initiatives have been instrumental in building the evidence base on violence against girls in developing countries, but these initiatives rarely examine conflicts and do not include younger adolescents [33,34]. Understanding predictors of violence in such settings is critical to developing effective strategies to prevent violence against adolescent girls.

We analyzed data from a baseline sample of displaced, conflict–affected adolescent girls in DRC and Ethiopia to assess the prevalence and related risk factors of physical, emotional, and sexual violence.

## METHODS

Eastern DRC has been in a state of constant conflict since 1996 and houses approximately 2.7 million internally displaced persons [35]. Ethiopia has been a primary destination for refugees fleeing protracted conflicts in Sudan and South Sudan, and as of September 2015, the area of Benishangul–Gumuz hosts an estimated 11 174 South Sudanese refugees [36].

This paper draws on data from a cross–sectional survey of violence and related risk factors for internally displaced and refugee adolescent girls between May–October 2015 in 14 sites across South Kivu, DRC, and in 3 refugee camps in Benishangul–Gumuz, Ethiopia. The survey was undertaken to obtain baseline information on girls’ experiences of violence prior to the implementation of an adolescent life skills and safe space program run by the International Rescue Committee in both countries [37]. In the DRC, adolescent girls were excluded from the survey if they were outside of the 10–14 age range, or lacked verbal proficiency in Swahili or Mashi. In Ethiopia, adolescent girls were excluded if they were outside the 13–19 age range, or lacked verbal proficiency in Funj, Regarig, Ingessana Kulelek, or Maban. Language exclusion was based on the selected languages spoken by most girls in the research settings and on language limitations of the data collectors (in the case of Ethiopia). Age exclusion was determined at country level based on age groups being targeted for programming. In both countries, participants with significant cognitive impairments or physical disabilities that would prevent independent completion of the questionnaire were excluded for ethical reasons.

Survey questionnaires that allowed for comparability were field–tested and used in both countries. In the DRC, a confidential survey, taking approximately one hour, was administered by data collectors matched to participants by gender and language. Less sensitive questions were administered using Computer–Assisted Personal Interview (CAPI), in which interviewers asked questions verbally and recorded responses on a tablet. Adolescent girls answered more sensitive questions on violence and sexual health using Audio Computer–Assisted Self–Interview (ACASI) programming, which allowed participants to listen to the questions and responses through headphones, and independently select responses. Girls aged 10–12 completed a modified survey, with questions deemed appropriate by community interlocutors and approved by local and Western ethics bodies; these girls were excluded from our analysis, as the modified survey excluded some of the predictors of interest. In Ethiopia, the entire survey was administered using ACASI, and there were no differences in survey administration based on age. CAPI was not used in Ethiopia because enrollment criteria included non–written languages. A detailed description of recruitment and enrollment methods can be found elsewhere [37].

One thousand two hundred and ninety–six adolescent girls aged 13–19 were included in our analysis. The sample includes girls in “early adolescence”, defined as age 10–14, and in “late adolescence”, defined as age 15–19 [38]. Based on an assumption of 15% prevalence of sexual violence and inclusion of up to 8 predictors in the model, a sample size of 170 would be needed both to determine prevalence and analyze a robust model, suggesting our sample in each country is sufficiently powered for this analysis.

### Ethics approval

All study procedures were approved by the Columbia University Institutional Review Board (IRB) and by in–country local bodies: the Ministry of Gender in DRC and the Administration for Refugee and Returnee Affairs in Ethiopia. Procedures undertaken to ensure confidentiality and mitigate the potential for harm included extensive training on ethics and consent, private spaces for interviews, use of ACASI for sensitive questions on violence victimization, and standardized debriefs that provided information about available psychosocial support services. Further details of our ethical protocols are detailed in our protocol paper [37].

### Analysis

Independent variables were selected for analysis based on known risk factors in non–humanitarian contexts and formative research on vulnerability in both countries. Variables were selected to include factors at the individual, relational, and communal levels of Michau et al’s adapted ecological model [13]. Independent variables included age, educational attainment, presence of biological parents in the home, living with an intimate partner, working without payment in the last 12 months, marital status, and ever having a boyfriend.

Outcome variables were chosen to include different forms of violence victimization, and included binary questions on physical, emotional, and sexual violence occurring in the last 12 months, adapted from ICAST and VACS questionnaires [34,39]. Physical violence was defined as being hit or beaten. Emotional violence was operationalized as being screamed at loudly or aggressively. Sexual violence was operationalized as experiencing unwilling (forced) sex, unwanted sexual touching, or verbal coercion (using influence or authority to threaten or pressure respondent to have sex). Independent relationships to violence outcomes were assessed using chi–square and Fisher exact tests, where appropriate. Models were analyzed using multivariable logistic regression, separated by country. All analyses were completed using STATA 13.1 (StataCorp LP, College Station, USA).

## RESULTS

### Sample demographics

**Figure 1:**
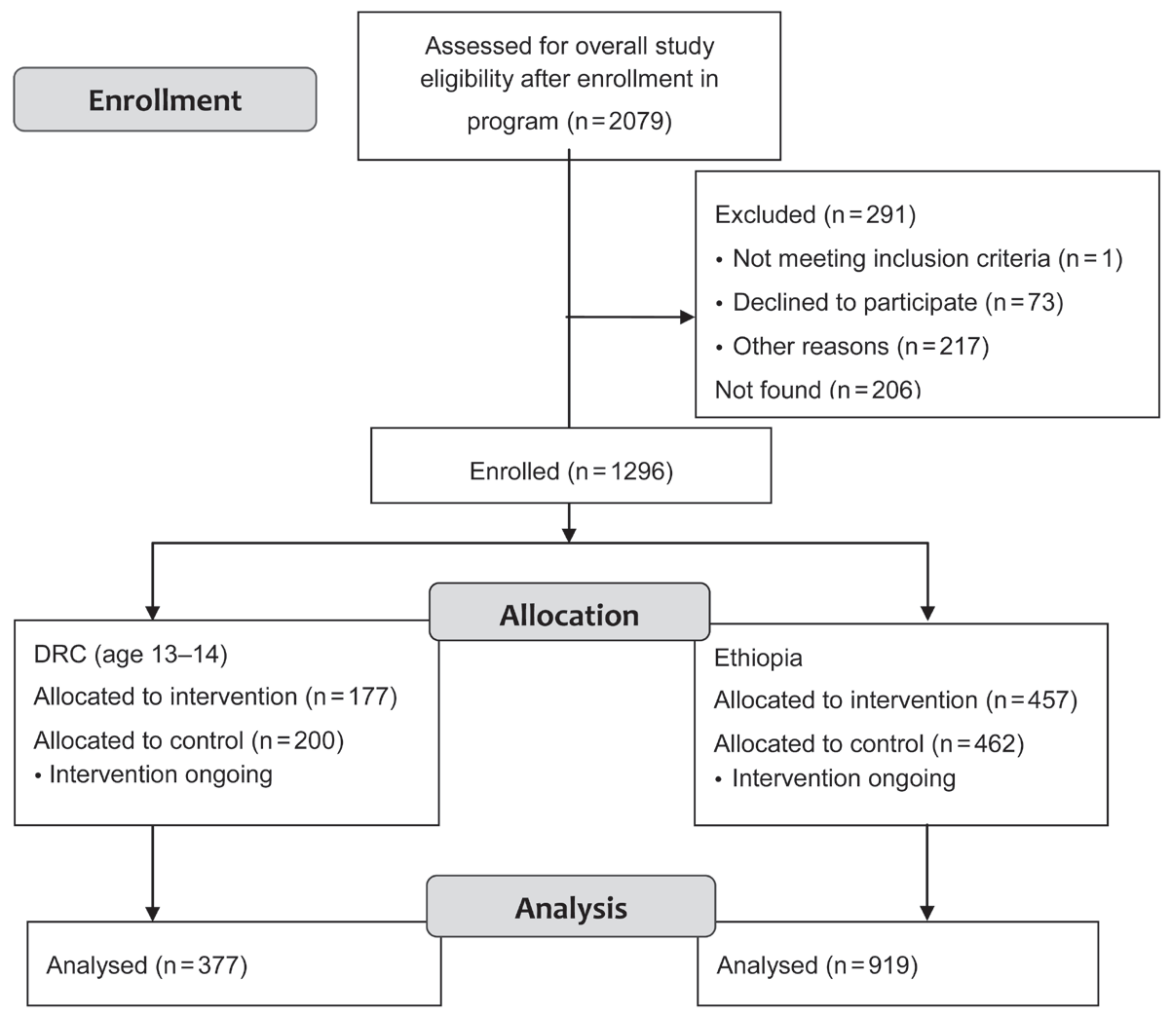
Participant flow diagram.

The sample included 1296 subjects aged 13–14 in DRC (N = 377) and aged 13–19 in Ethiopia (N = 919) (**Figure 1**). Mean age was 13.53 (standard deviation (SD) 0.50) years in DRC, and 14.61 (SD 1.51) years in Ethiopia. In DRC, 82.2% of participants had ever attended school, and in Ethiopia, 69.3% had ever attended. On average, participants had completed 4.37 (SD 2.26) years of school in DRC and 2.81 (SD 1.93) years in Ethiopia. In DRC, the most frequently reported reason for non–enrollment in school was financial difficulty in paying for school or associated costs (92.0%), while domestic responsibilities were the most frequent reason for non–enrollment in Ethiopia (27.6%). Marriage or pregnancy was reported as a greater barrier to school attendance in Ethiopia (16.3%) than in DRC (0.0%).

The vast majority of participants reported living with at least one biological parent in both settings (**Table 1**). Of those who provided information on marital status, 23.1% in DRC and 32.6% in Ethiopia reported being either married or living with someone as if married. Adolescent girls who were aged 18 or above were not significantly more likely to be married or living with someone as if married than adolescents aged 13–17 (*P* = 0.136). Approximately 17.7% of participants in DRC, and 24.5% in Ethiopia, reported living with an intimate partner. 20.7% (n = 78) of respondents in DRC, and 30.6% in Ethiopia, reported ever having a boyfriend.

**Table 1:** Demographic characteristics

	DRC (N = 377)		Ethiopia (N = 919)	
	**No.**	**%**	**No.**	**%**
**Marital status:**	(N = 350)		(N = 826)	
Unmarried	263	75.14	527	63.80
Married and living with partner	46	13.14	149	18.04
Married and not living with partner	25	7.14	97	11.74
Living with partner as if married	16	4.57	53	6.42
**Family structure:**	(N = 377)		(N = 880)	
Living with both parents	230	61.01	395	44.89
Living with mother only	104	27.59	240	27.27
Living with father only	12	3.18	173	19.66
Living with neither parent	31	8.22	72	8.18
**Education:**	(N = 377)		(N = 890)	
Ever attended school	310	82.23	637	71.57
Enrolled in school in last school year	160	51.61	502	81.76
**Work outside the home:**	(N = 377)		(N = 919)	
Worked without pay in the last 12 months	53	14.06	846	92.06

DRC – Democratic Republic of the Congo

### Prevalence of violence

Approximately half of the adolescent girls in our sample (54.4% in DRC, 50.5% in Ethiopia) reported experiencing at least one form of violence victimization in the previous 12 months. Of those who reported experiencing at least one form of violence, 48.3% in DRC and 49.1% in Ethiopia reported poly–victimization. As shown in **Table 2**, the most frequently reported form of violence in the last 12 months across both countries was emotional abuse. Younger adolescent girls (aged 13–14) in Ethiopia reported experiencing significantly more physical (odds ratio (OR) = 1.37, *P* = 0.037) and emotional violence (OR = 1.44, *P* = 0.012) compared with older girls (aged 15-19).

**Table 2:** Prevalence of physical, emotional, and sexual violence

	DRC		Ethiopia	
	**No.**	**%**	**No.**	**%**
**Physical violence:**	(N = 355)		(N = 850)	
Beaten or hit in last 12 months	124	34.93	259	30.47
**Emotional abuse:**	(N = 346)		(N = 839)	
Screamed at loudly or aggressively in last 12 months	133	38.44	303	36.11
**Sexual abuse:**	(N = 337)		(N = 821)	
Ever experienced forced sex	71	21.07	148	17.87
	(N = 369)		(N = 914)	
Experienced forced sex in last 12 months	58	15.72	128	14.00
	(N = 349)		(N = 839)	
Ever experienced unwanted sexual touching	69	19.77	201	23.96
	(N = 345)		(N = 828)	
Experienced unwanted sexual touching in last 12 months	38	11.01	108	13.04
	(N = 350)		(N = 824)	
Ever experienced threats or verbal coercion	62	17.71	251	30.46
	(N = 345)		(N = 808)	
Experienced threats or verbal coercion in last 12 months	30	8.70	96	11.88

DRC – Democratic Republic of the Congo

Approximately one–fourth of adolescent girls reported at least one type of sexual violence victimization within the previous 12 months (25.5% in DRC, 27.2% in Ethiopia). Forced sex was the most frequently reported form of sexual violence (**Table 2**). Again, younger girls (aged 13–14) in Ethiopia were 2.09 times more likely than older adolescents (aged 15–19) to report unwanted sexual touching (95% CI 1.37–3.20, *P* = 0.001), and 1.77 times more likely to report forced sex (95% CI 1.20–2.61, *P* = 0.004) in the previous 12 months.

The vast majority of adolescent girls reported that their intimate partners (boyfriends or husbands) and/or other family members (parents, caregivers or other relatives) were perpetrators of violence. Intimate partners were the most frequently reported perpetrator of violence for nearly all forms of violence in both countries, followed closely by caregivers or relatives (**Table 3**). In DRC, less than 10% of adolescent girls who reported physical, verbal, or sexual abuse reported that perpetrators were members of an armed group and/or officials with authority in the community. In Ethiopia, closer to 15% of adolescent girls reported members of an armed group and/or officials as perpetrators of physical, emotional, and sexual abuse (**Table 3**).

**Table 3:** Perpetrators of physical, emotional, and sexual violence

	DRC		Ethiopia	
	**No.**	**%**	**No.**	**%**
**Physical violence:**	(N = 124)		(N = 259)	
Boyfriend or husband	37	29.84	105	40.54
Parent, caregiver or other relative	58	46.77	68	26.25
Friend or neighbor	18	14.52	33	12.74
Member of an armed group	1	0.81	14	5.41
Official	2	1.61	20	7.72
Other	8	6.45	28	10.81
**Verbal abuse – loud or aggressive screaming:**	(N = 133)		(N = 303)	
Boyfriend or husband	40	30.08	97	32.01
Parent, caregiver or other relative	69	51.88	117	38.61
Friend or neighbor	10	7.52	49	16.17
Member of an armed group	2	1.50	16	5.28
Official	6	4.51	16	5.28
Other	8	6.02	22	7.26
**Unwanted sexual touching:**	(N = 69)		(N = 201)	
Boyfriend or husband	38	55.07	86	42.79
Parent, caregiver or other relative	18	26.09	58	28.86
Friend or neighbor	10	14.49	17	8.46
Member of an armed group	0	0.00	13	6.47
Official	0	0.00	16	7.96
Other	3	4.35	19	9.45
**Coerced sex:**	(N = 62)		(N = 251)	
Boyfriend or husband	31	50.00	93	37.05
Parent, caregiver or other relative	14	22.58	61	24.30
Friend or neighbor	11	17.74	41	16.33
Member of an armed group	2	3.23	22	8.76
Official	1	1.61	19	7.57
Other	5	8.06	26	10.36

### Predictors of violence

Because the participants in DRC and Ethiopia are known to have different demographic characteristics (mean age, ethnic group, legal status in country of residence), hypothesized predictors were first assessed for independent relationships to outcomes in the DRC and Ethiopia populations. To obtain a parsimonious model, predictors that were too closely related to other predictors were excluded from the model. Age, family structure, educational attainment, presence of biological parents in the home, living with a romantic partner, working without pay in the last 12 months, and ever having a boyfriend, were independently associated with violence outcomes in DRC and Ethiopia, and placed into an adjusted model to examine against each violence outcome in each country. The odds ratio, standard error, and 95% confidence interval for each model are shown in **Table 4** (DRC) and **Table 5** (Ethiopia).

**Table 4:** Prediction of adolescent-reported violence victimization in adjusted model, Democratic Republic of the Congo*

Predictor	Physical violence aOR [95% CI]	Emotional violence aOR [95% CI]	Any form of sexual violence aOR [95% CI]	Forced sex aOR [95% CI]	Unwanted sexual touching aOR [95% CI]	Coerced sex aOR [95% CI]
Age	0.684 [0.42,1.12]	0.826 [0.51,1.35]	0.818 [0.45, 1.48]	0.521 [0.26, 1.02]	1.085 [0.51, 2.31]	0.948 [0.42, 2.13]
Living with biological parents						
Living with mother only	1.522 [0.87, 2.66]	0.964 [0.56, 1.67]	1.158 [0.59, 2.28]	1.383 [0.64, 2.98]	0.714 [0.28, 1.79]	1.544 [0.65, 3.69]
Living with father only	2.224 [0.55, 8.93]	1.56 [0.39, 6.18]	1.318 [0.24, 7.24]	1.155 [0.13, 10.66]	1.277 [0.14, 11.31]	1.687 [0.19, 14.96]
Living with neither parent	1.308 [0.53, 3.24]	2.514 [1.01, 6.26]†	[0.31, 2.72] 0.918	0.939 [0.28, 3.13]	0.839 [0.21, 3.32]	0.302 [0.04, 2.50]
Years of school completed	0.897 [0.81, 0.99]†	0.948 [0.86, 1.04]	0.899 [0.80, 1.01]	0.851 [0.74,0.98]†	1.005 [0.87, 1.16]	0.998 [0.85, 1.17]
Living with intimate partner	1.418 [0.75, 2.67]	0.84 [0.43, 1.62]	2.899 [1.43, 5.88]†	2.696 [1.28, 5.69]†	2.105 [0.90, 4.90]	3.582 [1.50, 8.56]‡
Worked without pay, last 12 months	1.126 [0.55, 2.31]	0.986 [0.49, 1.98]	2.058 [0.91, 4.65]	0.892 [0.33, 2.40]	0.972 [0.33, 2.83]	1.772 [0.63, 4.98]
Ever had a boyfriend	2.963 [1.69, 5.20]§	2.891 [1.64, 5.09]§	6.368 [3.43, 11.82]§	8.657 [4.40, 17.03]§	4.099 [1.90, 8.85]§	2.323 [0.99, 5.44]
Observations	313	308	300	320	310	304

aOR – adjusted odds ratio, CI – confidence interval

*95% confidence intervals in brackets.

†*P* < 0.05.

‡*P* < 0.01.

§*P* < 0.001.

**Table 5:** Prediction of adolescent-reported violence victimization in adjusted model, Ethiopia*

Predictor	Physical violence aOR [95% CI]	Emotional violence aOR [95% CI]	Any form of sexual violence aOR [95% CI]	Forced sex aOR [95% CI]	Unwanted sexual touching aOR [95% CI]	Coerced sex aOR [95% CI]
Age	0.881 [0.78, 0.99]†	0.856 [0.77, 0.96]‡	0.902 [0.80, 1.02]	0.835 [0.71, 0.98]†	0.843 [0.72, 0.99]†	0.99 [0.85, 1.16]
Living with biological parents						
Living with mother only	1.051 [0.68, 1.63]	1.44 [0.97, 2.14]	1.216 [0.77, 1.91]	1.41 [0.82, 2.43]	1.013 [0.59, 1.75]	1.401 [0.79, 2.48]
Living with father only	2.115 [1.35, 3.32]‡	1.419 [0.93, 2.17]	2.008 [1.25, 3.22]‡	2.502 [1.46, 4.28]§	1.438 [0.83, 2.50]	1.477 [0.80, 2.74]
Living with neither parent	1.829 [0.93, 3.59]	1.125 [0.58, 2.16]	0.849 [0.37, 1.96]	0.443 [0.13, 1.54]	0.553 [0.18, 1.68]	1.046 [0.37, 2.95]
Years of school completed	0.946 [0.86, 1.04]	1.034 [0.95, 1.12]	0.927 [0.84, 1.02]	0.986 [0.88, 1.10]	0.948 [0.84, 1.07]	0.879 [0.77, 1.00]
Living with intimate partner	0.752 [0.49, 1.15]	1.149 [0.78, 1.69]	1.830 [1.20, 2.79]‡	1.28 [0.78, 2.11]	1.355 [0.82, 2.24]	1.443 [0.85, 2.45]
Worked without pay, last 12 months	2.324 [0.83, 6.49]	2.788 [1.11, 7.00]†	2.848 [0.80, 10.09]	2.339 [0.52, 10.45]	4.394 [0.57, 33.60]	1.00 [1.00, 1.00]
Ever had a boyfriend	4.504 [3.16, 6.41]§	2.784 [1.99, 3.89]§	4.655 [3.21, 6.76]§	4.577 [2.94, 7.13]§	4.295 [2.74, 6.74]§	4.153 [2.57, 6.70]§
Observations	697	690	663	723	692	630

aOR – adjusted odds ratio, CI – confidence interval

*95% confidence intervals in brackets.

†*P* < 0.05.

‡*P* < 0.01.

§*P* < 0.001.

In examining physical violence in our adjusted models, adolescent girls in both countries who had ever had a boyfriend were significantly more likely to disclose physical violence in the previous 12 months than those who had never had a boyfriend (DRC adjusted OR (aOR) = 2.96, *P* < 0.001; Ethiopia aOR = 4.50, *P* < 0.001). In DRC alone, each additional year of school completed was associated with 0.90 lower odds of victimization (*P* = 0.028). In Ethiopia, adolescent girls living with their father had 2.12 greater odds of disclosing physical violence in the previous 12 months, compared to those living with both biological parents (*P* = 0.001). Further, each additional year of age was associated with reduced odds of physical violence in Ethiopia (aOR = 0.88, *P* = 0.038).

For emotional abuse, adolescent girls in both countries who had ever had a boyfriend had greater odds of reporting exposure to loud and aggressive screaming in the previous 12 months compared with those who had never had a boyfriend (DRC aOR = 2.89, *P* < 0.001; Ethiopia aOR = 2.78, *P* < 0.001). In DRC, living with neither parent was marginally associated with greater odds of victimization, compared to living with both parents (aOR = 2.51, *P* = 0.048). In Ethiopia, each additional year of age was associated with a 0.86 reduced odds in reported exposure to loud and aggressive screaming (*P* = 0.006). Additionally, working without payment during the previous 12 months was associated with 2.79 greater odds in reported exposure to loud and aggressive screaming in Ethiopia (*P* = 0.029).

Sexual violence was examined in relation to experiencing unwanted sexual touching, experiencing forced sex, or experiencing coerced sex through influence, authority, threats or pressure in the previous 12 months. Living with an intimate partner was associated with higher odds of experiencing any form of sexual violence in the previous 12 months in both countries, compared to not living with an intimate partner (DRC aOR = 2.90, *P* = 0.003; Ethiopia aOR = 1.83, *P* = 0.005). Adolescent girls who had ever had a boyfriend also reported higher odds of experiencing any form of sexual violence in both countries, compared to those who had not had a boyfriend (DRC aOR = 6.37, *P* < 0.001; Ethiopia aOR = 4.66, *P* < 0.001). In Ethiopia, adolescent girls who reported living with their father had 2.01 times higher odds of experiencing any form of sexual violence than adolescents who were living with both biological parents (*P* = 0.004).

Having had a boyfriend was significantly associated with forced sex in both countries, when adjusting for other variables (see **Table 4** and **Table 5**). In DRC, living with an intimate partner was associated with 2.70 higher odds of forced sex (*P* = 0.009). Further, each additional year of school completed was also associated with 0.85 lower odds of forced sex (*P* = 0.022), and each additional year of age was marginally associated with 0.52 lower odds of forced sex (*P* = 0.059). In Ethiopia, each increased year of age was associated with 0.84 lower odds of forced sex (*P* = 0.027). Adolescent girls in Ethiopia who reported living with their father were 2.50 times more likely to report forced sex in the previous 12 months than adolescents living with both parents (*P* = 0.001).

Having had a boyfriend was similarly associated with greater odds of unwanted sexual touching victimization in the previous 12 months in both countries, compared with adolescent girls who reported never having had a boyfriend (DRC aOR = 4.10, *P* < 0.001; Ethiopia aOR = 4.30, *P* < 0.001). In Ethiopia, increased age was associated with lower odds of reported unwanted sexual touching (aOR = 0.84, *P* = 0.037).

Finally, experiencing coerced sex through influence, authority, threats or pressure in the previous 12 months was associated with living with an intimate partner in DRC (aOR = 3.58, *P* = 0.004), and ever having a boyfriend in Ethiopia (aOR = 4.15, *P* < 0.001). Ever having a boyfriend was also marginally associated with coerced sex in DRC (aOR = 2.32, *P* = 0.052). Each additional year of completed education was marginally associated with lower odds of coerced sex in Ethiopia (aOR = 0.88, *P* = 0.059).

## DISCUSSION

The prevalence of physical, emotional, and/or sexual violence victimization among adolescent girls (51.62%) is similar to the regional prevalence of past–year violence estimated from census data of girls and boys aged 2–14 (50%) and 15–17 (51%) in Africa [33].

Our study broadens the limited evidence base on predictors of violence against conflict–affected adolescent girls. The fact that early age was associated with increased odds of physical, emotional, and most measured forms of sexual violence in Ethiopia is concerning, especially in a sample that interviewed girls in early adolescence. We also found a significant association between years of education completed and violence victimization among displaced girls aged 13–14 in the DRC, when adjusting for other factors [12]. Although age and educational attainment are commonly considered to be risk factors for victimization in our contexts, our findings demonstrate that the relationship between these variables and victimization may not be consistent across conflict–affected populations in the East Africa region.

Caregivers and other family members tended to perpetrate much of the reported violence against adolescent girls. Similarly, ever having a boyfriend was the most consistent predictor of sexual violence, even when adjusting for risk factors known to be associated with violence in other contexts. These findings suggest that engagement in intimate relationships may be *the* primary risk factor for violence victimization among adolescents aged 13–19 in conflict settings.

Importantly, predictors of different forms of sexual violence were not uniform, suggesting that adolescent girls’ vulnerability to victimization differs across forms of sexual violence and contexts. While living with an intimate partner was associated with coerced and forced sex in DRC for example, it was not significantly associated with unwanted sexual touching in DRC, or with any of the three forms of sexual violence in Ethiopia.

Finally, our findings indicate that living only with one’s father is a predictor for both physical and sexual violence in Ethiopia, but not DRC. While high–income countries have also documented increased risk of victimization for girls living with a single parent, the majority of single parents in developed contexts are mothers [14,15]. In our study settings, the particular pathways through which increased vulnerability occurs are unclear, but the absence of the mother in the home increased the vulnerability of adolescent girls in Ethiopia. These findings suggest contextual differences in household makeup that should be further explored.

Taken together, our results have important implications for gender–based violence prevention efforts in conflict settings. Increased risk of violence among younger adolescents indicates that prevention efforts must target younger populations, who may be at increased risk of victimization.

Our findings contradict narratives suggesting that girls are most at risk of violence at the hands of strangers or military personnel in conflict settings [24]. These findings also call into question local narratives positing that early marriage is “protective” in conflict and helps keep girls safe from violence, or preserves a family’s “honor” [40,41]. Considering the evidence from our study and others on intimate partners as primary perpetrators of violence, even in humanitarian emergencies [42], prevention programming should focus on explicitly acknowledging the presence of intimate partner relationships, even in more culturally conservative settings. Our findings suggest that humanitarian programming may need to include intimate partner violence prevention strategies for adolescent girls, and work with adult community members to understand how some practices intended to “protect” girls, such as early marriage, may put girls at a greater risk for violence.

Caregivers were also identified as primary perpetrators, which supports existing calls to target key adults in adolescent girls’ lives, such as caregivers and other relatives, and to support positive parenting strategies and communication with adolescent girls [43,44]. Such parenting programs should address the additional vulnerabilities that girls may experience in conflict settings. While further research is warranted, our findings suggest that male caregivers should in particular be targeted for primary prevention efforts in conflict settings.

Finally, our study has implications for efforts to document violence against adolescent girls in humanitarian settings. Considering that more than 30% of adolescent girls in our study had not attended school in the previous year, researchers should use school–based sampling methodologies with caution.

Limitations of this study include the fact that single indicators were employed to measure physical and emotional violence, and potentially relevant predictors were not included in the survey such as length of displacement, previous exposure to war–related violence, alcohol consumption or drug use, and caregiver exposure to violence. Although interview methodologies were informed by constraints of the languages of participants, it is possible that use of different methodologies (both CAPI and ACASI in DRC, and solely ACASI in Ethiopia) may have contributed to some differences in predictors identified in the two contexts examined. To account for limitations imposed by the cross–sectional survey design, we restricted independent variables to relationships for which temporality could be reasonably inferred. For example, information on adolescents’ self–esteem was excluded from our model because researchers could not determine temporality of self–esteem and reported violence. Finally, the study is limited to adolescent girls and caregivers who self–selected to join the life skills program. To increase awareness of the program, recruitment efforts included community sensitization to the program, which were conducted throughout the villages in South Kivu and camps in Ethiopia. Even so, those adolescents who may be most marginalized and who lack the access and social capital to join the program were likely underrepresented in this study.

## CONCLUSION

Globally, females are at highest risk for violence during adolescence, and humanitarian emergencies may exacerbate these vulnerabilities. Persistent gaps in knowledge of violence victimization have, to date, limited the humanitarian community’s ability to appropriately respond to and prevent violence against adolescent girls in these contexts. Our study sheds light on prevalence and predictors of violence for conflict–affected adolescents aged 13–19 in two contexts, and offers important evidence for targeted programming and policy response for emergency actors, as well as guidance for other researchers working in these settings.
